# Parent–child associations for changes in diet, screen time, and physical activity across two decades in modernizing China: China Health and Nutrition Survey 1991–2009

**DOI:** 10.1186/s12966-016-0445-z

**Published:** 2016-11-11

**Authors:** Fei Dong, Annie Green Howard, Amy H. Herring, Amanda L. Thompson, Linda S. Adair, Barry M. Popkin, Allison E. Aiello, Bing Zhang, Penny Gordon-Larsen

**Affiliations:** 1 Department of Nutrition, Gillings School of Global Public Health & School of Medicine, University of North Carolina at Chapel Hill, Chapel Hill, NC USA; 2 Department of Biostatistics, Gillings School of Global Public Health, University of North Carolina at Chapel Hill, Chapel Hill, USA; 3 Carolina Population Center, University of North Carolina at Chapel Hill, 137 East Franklin Street, Room 6124, Campus Box #8120, Chapel Hill, NC 27514 USA; 4 Department of Anthropology, University of North Carolina at Chapel Hill, Chapel Hill, USA; 5 Department of Epidemiology, Gillings School of Global Public Health, University of North Carolina at Chapel Hill, Chapel Hill, USA; 6 National Institute for Nutrition and Health, Chinese Center for Disease Control and Prevention, Beijing, China

**Keywords:** Animal-source food, Away-from-home eating, Snacking, Screen time, Leisure-time sports, Household structure, Urbanization, China

## Abstract

**Background:**

While the household context is important for lifestyle behavior interventions, few studies have examined parent–child associations for diet and physical activity (PA) changes over time in a rapidly urbanizing country. We aimed to investigate changes in diet, screen time, and PA behaviors over time in children and their parents living in the same household, and examine the parent–child association for these behaviors.

**Methods:**

We studied dietary, screen time, and PA behaviors in 5,201 parent–child pairs (children aged 7-17y) using longitudinal data from the China Health and Nutrition Survey (1991, 1993, 1997, 2000, 2004, 2006, and 2009). We collected three-day 24-h recall diet data to generate percentages of energy from animal-source foods, away-from-home eating, and snacking from 1991–2009, which are known urbanization-related behaviors. We used a seven-day PA recall to collect screen time (hours/week) and leisure-time sports participation (yes/no) since 2004. We examined the changes in children’s and parents’ behaviors over time using random-effects negative binomial regression for diet and screen time, and random-effects logistic regression for leisure-time sports. We then regressed each of the behaviors of offspring on each of their parents’ same behaviors to examine the parent–child association, using the same set of models.

**Results:**

We observed increases in energy from animal-source foods, eating away-from-home, and snacking, as well as screen time and leisure-time sports in parents and children over time, with different rates of change between children and their parents for some behaviors. We found positive parent–child associations for diet, screen time, and PA. When parental intakes increased by 10 % energy from each dietary behavior, children’s increase in intakes ranged from 0.44 to 1.59 % total energy for animal-source foods, 0.17 % to 0.45 % for away-from-home eating, and 2.13 % to 7.21 % for snacking. Children were also more likely to participate in leisure-time sports if their parents participated in leisure-time sports.

**Conclusion:**

Our findings support household-based health behavior interventions targeting both children and their parents. However, generation-specific intervention strategies may be needed for children and adults, especially for dietary behaviors, which changed differentially in children versus parents in this rapidly modernizing population.

**Electronic supplementary material:**

The online version of this article (doi:10.1186/s12966-016-0445-z) contains supplementary material, which is available to authorized users.

## Background

The prevalence of obesity [1] and cardiometabolic disease risk factors [2] has been burgeoning over the past two decades in China among both adults and children, with a faster increase in children [3]. Dietary and physical activity (PA) behaviors are important contributors to obesity [4] and cardiometabolic health [5, 6]. Thus, understanding factors that influence these behaviors can be essential for designing successful interventions. Family indisputably plays an important role in shaping health-related behaviors of the household unit. Children’s diet and PA behaviors have been shown to be associated with those of their parents [7–13]. This is possibly because parents may share meals and participate in PA with their offspring and can serve as role models and influence children’s preferences and attitudes towards diet and PA. However, in the face of environmental change, such as that seen in China with modernization, rates of change in childhood eating behaviors have been shown to differ from those of adults [14].

Past research on the parent-offspring association for diet and PA, however, is mostly cross-sectional. These studies were unable to examine the change of diet and PA behaviors over time, especially in rapidly modernizing populations such as China. Evaluating and comparing changes in diet and PA behaviors in children and their parents is essential to understand whether urbanization influences children and adults differently and to track whether changes in children mirror those of their parents. A few longitudinal studies have investigated the parent–child association for diet and PA behaviors [8–10], and found statistically significant aggregation of behaviors between children and their parents. However, these studies have focused on high income countries [10] or only examined the mother-child association [8, 9]. Besides the influence of parents, the presence of grandparents and siblings may also play a role in children’s health behaviors, possibly because grandparents are more likely to indulge their grandchildren with modern, unhealthy foods and screen-based sedentary behaviors, especially in single-child households [15–17]. Nonetheless, to our knowledge, no research has examined how household structure (i.e. living with grandparents or not, having siblings or not) relates to children’s diet and PA behaviors in large Chinese population-based studies.

To address these gaps, we used longitudinal data from children and their parents enrolled in the China Health and Nutrition Survey (CHNS) from 1991 to 2009 to study changes in urbanization-related diet, screen time, and PA behaviors over time in children compared to their parents. We also examined whether children’s diet, screen time, and PA behaviors associated with those of their parents, and whether these behaviors in children differed by household structure.

## Methods

### CHNS

The CHNS is a household-based longitudinal cohort study with ongoing data collection in 228 communities across nine provinces throughout China (North: Heilongjiang, Liaoning; Central: Shandong, Henan, Jiangsu; South: Hunan, Hubei, Guangxi, Guizhou) in nine survey rounds from 1989–2011. Using a multistage, random cluster design, a stratified probability sample was used to select counties and cities stratified by income using State Statistical Office definitions [18]. Communities and households were then selected from these strata. We used questionnaires to collect demographic, socioeconomic, behavioral, and health information from each household member. The CHNS cohort initially mirrored national age-sex-education profiles [19–21] and the provinces in the CHNS sample constituted 44 % of China’s population in 2009 (according to 2009 census). More details on the survey procedures are described previously [22]. The study was approved by the Institutional Review Board at the University of North Carolina at Chapel Hill, the China-Japan Friendship Hospital, Ministry of Health, and the National Institute for Nutrition and Health, Chinese Center for Disease Control and Prevention. Subjects gave informed consent for participation.

### Analysis sample

We used longitudinal data from 1991, 1993, 1997, 2000, 2004, 2006, and 2009 when data on parents and children were collected. Eligible subjects were parent–child pairs living in the same household with children aged 7-17y at any exam period who had at least two waves of diet, screen time, and PA data (*n* = 5,287). Due to the age restriction, participants aged in and out of our sample at different years during the 18-year follow up, thus at each year there was a mixture of different groups of parent–child pairs with children aged 7–17y followed for 2–4 surveys instead of one group of parent–child pairs who were followed over time. We excluded parent–child pairs with missing covariates (*n* = 86). Our final analytic sample included 5,201 unique parent–child pairs with data across the study period (5,151 mother-child and 5,091 father-child pairs; 4,979 households had all three members) with an average of 2.4 visits for diet behaviors; 2.3 visits for screen time and PA behaviors. Compared to the excluded parent–child pairs, children included in the analytic sample were more likely to have lower household income, to live in less urbanized areas, to have parents with lower education level, and to fit the age criteria in earlier survey years (Additional file 1: Table S1). Included children also had lower mean values for animal source-foods, away-from home eating, and screen time relative to excluded children.

### Diet

We focused on three dietary behaviors that most reflect urbanization: animal-source food intake, away-from-home food consumption, and snacking [23]. Our diet data were derived from three consecutive 24-h dietary recalls at the individual level and a food inventory at the household level occurring during the same 3-day period, randomly starting from Monday to Sunday. All foods available in the household were measured on a daily basis for the food inventory. For the 24-h recalls, trained interviewers recorded types and amounts of foods, types of meal, and places of food preparation of all food items consumed by each household member. For children younger than 10y, mothers or mother substitutes were asked to recall children’s dietary intake. The energy content of foods was based on a Chinese food composition table [24]. This dietary assessment has been validated relative to doubly labeled water (r^2^ men: 0.56; women: 0.60) for energy [25] and urine for sodium (r^2^: 0.58), potassium (r^2^: 0.59), and MSG (r^2^: 0.82) [26]. In this study we focused on three dietary behaviors that most reflect urbanization: animal-source food intake, away-from-home food consumption, and snacking [23]. Animal-source foods included meats and meat products, eggs and egg products, fish and seafood, milk and dairy products. We defined away-from-home foods as foods prepared away from home (no matter if they were consumed at or away from home) and snacks were reported as foods consumed outside the three main meals (breakfast, lunch and dinner).

### Physical activity (PA)

Our PA data were derived from seven-day PA recalls across a variety of domains. Children and parents were asked about their participation and time spent in different types of sedentary behavior and PA. Parents or primary caregivers completed or assisted with completing the surveys for children under 10y. The PA components have been found to be highly predictive of incident obesity and weight gain in adults [27–30]. In this study we examined screen time (hours/week) and leisure-time sports participation (yes/no). Screen time referred to time spent on TV/videotape watching, video games, and computer usage. Leisure-time sports included gymnastics, dancing, track and field sports, swimming, ball sports (e.g. basketball, tennis), and other sports (e.g., martial arts, tai chi). We focused on children’s leisure-time sports outside school only (including before or after school for those in school and any activity for those not in school) for the purpose of studying the parent–child correlation, since in-school sports participation is mostly influenced by school instead of parents. Due to the low participation rate in leisure-time sports in both parents and children in all survey years, we dichotomized the variable into any versus no participation. Since data on screen time in adults were first collected in 2004 and the leisure time sports survey changed in 2004, we restricted our analyses for these behaviors to 2004, 2006, and 2009 only.

### Covariates

We collected participants’ age (y) at baseline (year when the child aged into the 7-17y range), sex, number of children in the household (1/>1), generation of family members in the household (2-generations: parents and children; 3-generations: grandparents, parents, and children), household income (inflated to 2011, tertiles), geographic region (North/Central/South), year of study entry, and highest parental education (none or primary/middle school/high school/technical, college or higher). We also used the CHNS multicomponent urbanicity scale comprised of 12 urban environment domains representing infrastructure, economic, and social service. The scale has high reliability and validity [31]. The scale ranges from 0–120 with a higher score reflecting higher urbanicity, which was categorized into year-specific tertiles.

### Statistical analysis

We conducted all analyses using Stata 14.0 (Stata Corp, College Station, TX, USA), and used a conservative *p*-value < 0.01 as our significance level due to our large sample size and the large number of statistical tests. In the descriptive analysis, we examined demographic characteristics of our analytic sample and tested differences of these characteristics over time using the chi-squared test (categorical variables) and one-way ANOVA (continuous variables).

For statistical models, we conducted random-effects negative binomial regression for our three dietary behaviors and screen time, with the random intercept for individuals. Three dietary variables are presented as the percentage of total energy (% energy) from these foods. Screen time is presented as hours per week. We used negative binomial models because the distribution of these variables was skewed and over-dispersed. We ran separate models for each behavior. For leisure-time sports, we used random-effects logistic regression to determine the change in the probability of participation in sports over time, accounting for repeated measures.

First, we examined the change in diet, screen time, and PA behaviors in children and their parents over time. Main exposures included year and dummy variables indicating household members (child, mother, father). To test whether the change in behaviors differed between children and their parents, we examined interactions between year and household members using the Wald test. We also tested if children’s change in behaviors differed by child’s baseline age and sex. Models controlled for sex and baseline age, time-varying household income and urbanicity, geographic region, and year of study entry. Then, we predicted adjusted mean values or adjusted probability of each behavior at each year by household member, with the year-household member interaction included in the models where the interaction was statistically significant.

Because participants aged in and out of our sample at different times during the 18-year follow up, at each wave there was a mixture of different groups of parent–child pairs with children aged 7-17y surveyed for 2–4 time points, instead of one identical group of parent–child pairs who were followed over time. To test whether changes in behaviors were similar for an identical group of parent–child pairs followed over time, we tested such differences in sensitivity analysis. Due to the age restriction of our sample, the maximum number of surveys a parent–child pair could complete was four. Therefore, in this sensitivity analysis we re-ran our dietary models among two separate groups of individuals who completed all four surveys. The first group of identical individuals was followed from 1991 to 2000 (*n* = 1,392), whereas the second was followed from 2000 to 2009 (*n* = 768). For screen time and PA, we re-ran our analyses among one group of individuals who completed all three surveys from 2004 to 2009 (*n* = 1,725).

Next, to determine the association between children’s behaviors with parental behaviors, we regressed each behavior of offspring on their parents’ same behavior, controlling for the same set of potential confounders. We ran separate models for mother-child and father-child pairs. For animal-source foods, away-from-home eating, and snacking, we predicted the change of children’s daily intake (% energy) when parental intake increased by 10 % total energy. For screen time, we predicted children’s change in screen time (hours/week) when parental screen time increased by one hour per week. We also estimated the odds ratios (OR) of leisure-time sports participation in children based on parental participation status. To examine whether the associations differed across years, we tested for interactions between parental behaviors with year. To further explore factors modifying the association, we examined the modification of association by sociodemographic factors including child’s sex and baseline age, household income, urbanicity, and geographic region. We predicted the associations with the interaction terms included in the models where the interactions were statistically significant.

Last, we examined whether children’s diet, screen time, and PA varied by household structure after adjusting for potential confounders. Main exposures were year and household structure variables (number of children in the household, generation of family members in the household). Then, we predicted adjusted mean values or adjusted probability of each behavior across all years by household structure using the models.

## Results

We observed increases in parental education level, household income, and urbanicity from 1991 to 2009 (*p* < 0.01; Table 1). The proportions of three-generation households and one-child households also increased over time.

**Table 1:** Characteristics of analytic sample over time, China Health and Nutrition Survey 1991–2009

	1991	2000	2009
No. of parent–child pairs	2257	2638	772
Child’s age, y (mean ± SD)^a^	12.6 ± 3.2	13.0 ± 2.9	14.2 ± 2.3
Mother’s age, y (mean ± SD)^a^	38.5 ± 5.4	39.1 ± 5.5	40.3 ± 4.4
Father’s age, y (mean ± SD)^a^	40.4 ± 6.0	40.5 ± 5.8	41.8 ± 5.0
Child’s gender, % male	51.4	52.7	53.2
Highest parental education, %^a^			
None/primary school	18.8	8.2	9.2
Middle school	28.4	18.5	22.8
High school	49.1	67.0	58.9
College, technical or higher	3.8	5.8	9.1
Number of generation, % three-generation^a, b^	26.1	33.1	57.9
Number of children, % one child^a^	47.2	45.9	72.4
Annual household income, 1000 yuan (mean ± SD)^a, c^	11.4 ± 8.2	19.2 ± 19.8	40.0 ± 40.0
Urbanicity (mean ± SD)^a, d^	42.4 ± 15.2	54.2 ± 16.9	61.6 ± 18.4

^a^Statistically different across years at the *p* < 0.01 level using one-way ANOVA (continuous variables) or chi-squared test (categorical variables)

^b^Three-generation: children, parents and grandparents (versus two-generation: children and parents)

^c^Total household income inflated to 2011

^d^Urbanicity defined by a multicomponent urbanicity scale ranging from 0–120 [31]

**Fig. 1:**
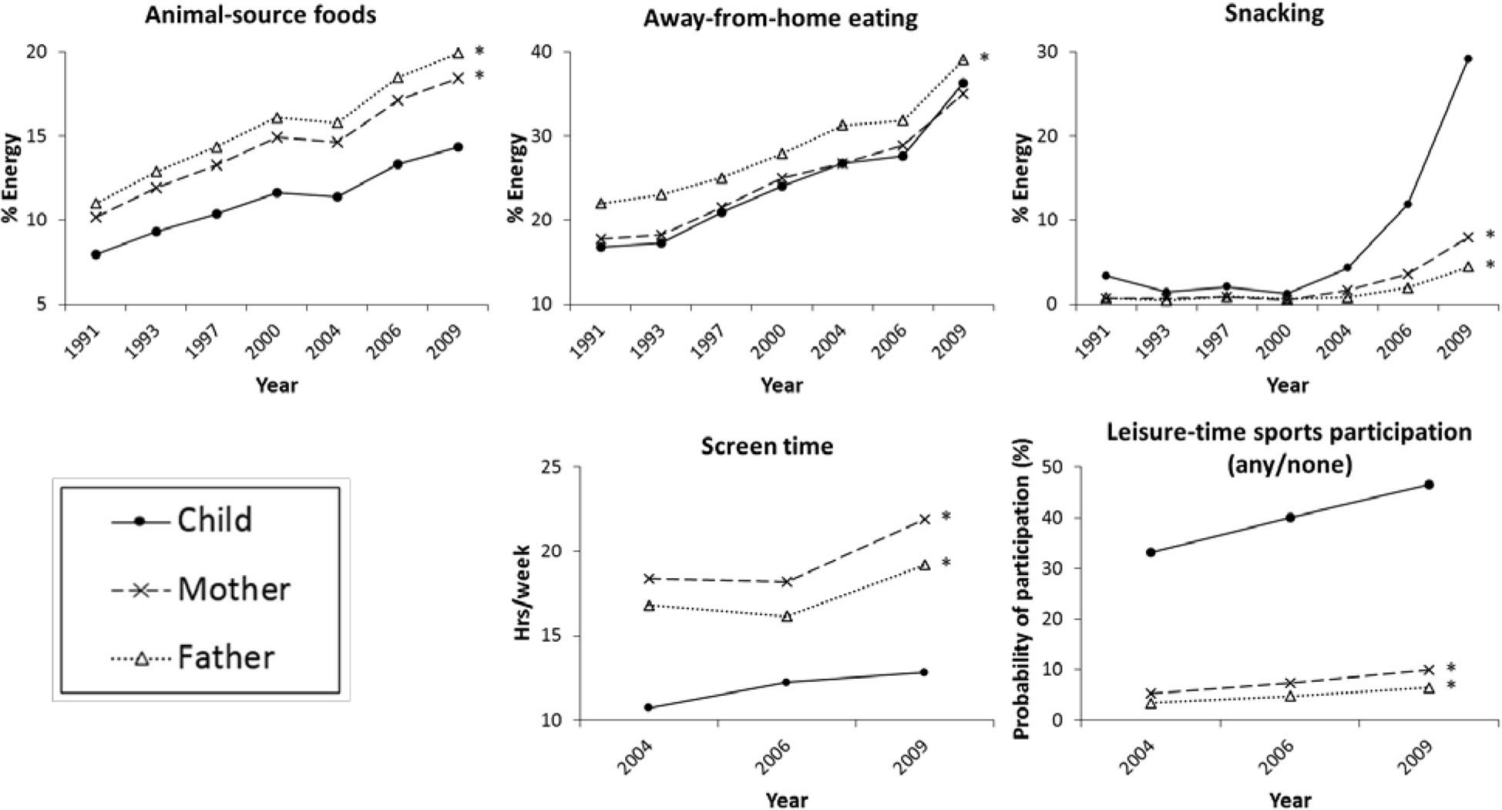
Predicted mean or probability of diet, screen time and PA among children, mothers, and fathers^a.^ ^a^Separate random-effects negative binomial regression models for each behavior predicted adjusted mean values of animal-source foods, away-from-home eating, snacking, and screen time; random-effects logistic regression model predicted adjusted probability of leisure-time sports participation. All models controlled for baseline age (y), household income (tertiles), urbanicity (tertiles), geographic region (North/Central/South), and year of study entry. The rate of changes in away-from-home eating, snacking, and screen time was different across household members (*p* for interaction < 0.01). PA, physical activity. *p < 0.01 comparing the mean dietary, screen time, and PA measures (across all years) between the starred parent and the child (the reference)

Model-adjusted predictions showed increasing percentages of energy from animal-source foods, away-from-home eating, and snacking in both children and their parents from 1991 to 2009 (Fig. 1; beta coefficients shown in Additional file 1: Table S2). Notably, the increase in animal-source foods slowed down in all household members from 2000 to 2004 before continuing after 2004, possibly due to a rapid increase in the price of meat during the same period of time, as shown in previous research [23]. The consumption of snacks started to increase dramatically after 2000, likely resulting from a major shift in food marketing related to snacking behaviors [32]. Compared to their parents, children consumed less energy from animal-source foods and away-from-home eating, but more energy from snacks. We detected statistically significant differences in the rate of change in behaviors for children compared to their parents for away-from-home eating and snacking (*p* for interaction < 0.01), indicating a faster increase of these behaviors in children versus adults in later years. Screen time and leisure-time sports participation increased in both children and their parents over time. Children spent less time on screen-based activities and were more likely to participate in leisure-time sports compared to their parents. Children’s screen time increased faster than their parents from 2004–2006 and slower than their parents from 2006–2009. We saw no differences in rates of change in the probability of leisure-time sports participation over time between children and their parents. Nor did we observe differences in change in these behaviors over time by child’s sex or baseline age.

Sensitivity analysis showed similar patterns of changes in animal-source foods and snacking among two groups of identical individuals followed from 1991 to 2000 and from 2000 to 2009, respectively (Additional file 1: Figure S1), compared to our central analysis. For eating away-from-home among individuals followed from 1991 to 2000 we observed faster increase among children than their parents, but the same rate for children and their parents from 2000 to 2009. For screen time, the rate of change did not differ across household members. There was no statistically significant change in the probability of leisure-time sports participation from 2004 to 2009 for all household members.

We found positive associations between children’s behaviors with those of their parents (Table 2). There was statistically significant effect measure modification by year for all behaviors except for leisure-time sports and by urbanicity for animal-source foods and away-from-home eating, so results are presented by year for all behaviors and by urbanicity where the interaction was statistically significant. We also detected statistically significant modification by geographic region and by household income for some behaviors. Due to the inclusion of multiple interactions in our models, we present the parent–child association by year and urbanicity at the medium income level in the Central region in Table 2. We saw no evidence of modification of the parent–child association in diet, screen time, and PA by sex or by child’s baseline age.

**Table 2:** Predicted parent-offspring associations for diet (% energy), screen time (hours/week), and leisure-time sports (any/none)^a^

	1991		1993		1997		2000		2004		2006		2009		P-interaction with year
	Beta	99 % CI	Beta	99 % CI	Beta	99 % CI	Beta	99 % CI	Beta	99 % CI	Beta	99 % CI	Beta	99 % CI	
Animal-source foods, % energy^b^															
Mother (*n* = 4264)															
Low urbanicity	1.59	1.50,1.67	1.51	1.43,1.58	1.43	1.36,1.51	1.27	1.19,1.34	1.28	1.20,1.36	1.19	1.10,1.28	1.14	1.03,1.25	<0.001
Medium urbanicity	1.26	1.19,1.34	1.18	1.11,1.25	1.11	1.04,1.18	0.94	0.88,1.01	0.96	0.88,1.03	0.87	0.79,0.95	0.82	0.71,0.92	
High urbanicity	0.89	0.81,0.97	0.81	0.74,0.88	0.74	0.67,0.81	0.57	0.51,0.64	0.58	0.51,0.66	0.49	0.41,0.57	0.44	0.34,0.55	
Father (*n* = 3906)															
Low urbanicity	1.44	1.35,1.53	1.37	1.28,1.45	1.30	1.22,1.38	1.20	1.11,1.28	1.17	1.08,1.27	1.12	1.02,1.22	1.07	0.95,1.19	<0.001
Medium urbanicity	1.20	1.11,1.29	1.13	1.05,1.21	1.06	0.98,1.14	0.96	0.88,1.04	0.93	0.85,1.02	0.88	0.79,0.98	0.83	0.71,0.94	
High urbanicity	0.87	0.78,0.96	0.80	0.72,0.87	0.73	0.65,0.81	0.63	0.55,0.71	0.60	0.51,0.69	0.55	0.46,0.64	0.49	0.38,0.61	
Away-from-home eating, % energy^b^															
Mother (*n* = 4280)															
Low urbanicity	0.45	0.41,0.49	0.43	0.40,0.47	0.41	0.37,0.44	0.35	0.31,0.38	0.33	0.30,0.37	0.35	0.31,0.39	0.29	0.25,0.33	<0.001
Medium urbanicity	0.41	0.37,0.44	0.39	0.36,0.42	0.37	0.33,0.40	0.31	0.28,0.34	0.29	0.26,0.32	0.31	0.27,0.34	0.25	0.21,0.28	
High urbanicity	0.33	0.29,0.36	0.31	0.28,0.35	0.29	0.26,0.32	0.23	0.20,0.26	0.21	0.18,0.24	0.23	0.20,0.26	0.17	0.13,0.20	
Father (*n* = 3917)	0.28	0.25,0.31	0.28	0.25,0.31	0.27	0.24,0.29	0.25	0.22,0.28	0.24	0.21,0.27	0.26	0.23,0.29	0.23	0.19,0.27	0.002
Snacking, % energy^b^															
Mother (*n* = 4276)	4.55	2.87,6.22	6.62	4.75,8.49	7.00	5.07,8.93	5.51	3.45,7.56	7.21	4.84,9.57	5.24	3.47,7.01	2.39	0.91,3.86	<0.001
Father (*n* = 3918)	4.49	2.59,6.39	7.17	4.66,9.67	4.88	2.90,6.86	3.44	1.49,5.39	6.59	3.41,9.77	4.15	1.81,6.49	2.13	0.17,4.08	<0.001
Screen time, hrs/week^c^															
Mother (*n* = 845)									0.02	0.01,0.03	0.01	0.00,0.02	0.01	0.00,0.02	0.002
Father (*n* = 742)	-	-	-	-	-	-	-	-	0.02	0.01,0.03	0.01	0.00,0.02	0.01	−0.00,0.01	0.009
Any leisure-time sports participation, %^d^															
									OR	99 % CI	OR	99 % CI	OR	99 % CI	
Mother (*n* = 817)	-	-	-	-	-	-	-	-	1.98	1.05,3.71	1.98	1.05,3.71	1.98	1.05,3.71	0.36
Father (*n* = 735)	-	-	-	-	-	-	-	-	2.83	1.52,5.28	2.83	1.52,5.28	2.83	1.52,5.28	0.99

Due to our large sample size, 99 % confidence intervals (CIs) were calculated instead of 95 % CIs to correspond to the p-values of 0.01

^a^Table shows coefficients at medium income in the Central region, and by urbanicity where there was statistically significant interaction by urbanicity. Separate random-effects negative binomial regression models for each behavior predicted beta coefficients for animal-source foods, away-from-home eating, snacking, and screen time; random-effects logistic regression model predicted odds ratios (OR) for leisure-time sports participation. All models controlled for child’s baseline age (y) and sex, household income (tertiles), urbanicity (tertiles), geographic region (North/Central/South), year of study entry, and highest parental education (none or primary/middle school/high school/technical, college or higher)

^b^Beta coefficients for animal-source foods, away-from-home eating, and snacking indicate the change of child’s daily intake in percentage of total energy with mother’s or father’s intake increased by 10 % total energy

^c^Beta coefficients for screen time indicate the change of child’s screen time in hours/week with mother’s or father’s screen time increased by one hour per week

^d^Parent-child association presented as ORs of participation in children based on parental participation status

Children’s dietary behaviors were positively associated with those of their parents, although in general the magnitude of associations declined over time. For example, at the medium urbanicity level, with 10 % increase in energy from animal-source foods in mothers, children’s intake increased by 1.26 % (99 % CI: 1.19-1.34) in 1991 and by 0.82 % (99 % CI: 0.71, 0.92) in 2009. For away-from-home eating, when maternal intake increased by 10 %, children’s intake increased by 0.41 % (99 % CI: 0.37, 0.44) in 1991 and by 0.25 % (99 % CI: 0.21, 0.28) in 2009. For snacking, with 10 % increase in maternal intake, children’s intake increased by 4.55 % (99 % CI: 2.87, 6.22) in 1991 and by 2.39 % (99 % CI: 0.91, 3.86) in 2009. For animal-source foods in both mother-child and father-child pairs, and for away-from-home eating in mother-child pairs, the parent-offspring associations were modified by urbanicity, as shown in Table 2. The associations were weaker with higher urbanicity levels. For instance, with 10 % increase in paternal intake of animal-source foods in 1991, children’s intake increased by 0.87 % (99 % CI: 0.78, 0.96) in high urbanicity areas, compared to by 1.44 % (99 % CI: 1.35, 1.53) in low urbanicity areas.

Similarly, children’s screen time and PA were positively associated with those of their parents. For example, with one hour/week increase in mother’s screen time, children’s screen time increased by 0.02 h/week (99 % CI: 0.02, 0.03) in 2004 and by 0.01 h/week (99 % CI: 0.01, 0.02) in 2009. Compared to children whose parents did not participate in leisure-time sports, children whose parents participated were more likely to participate in leisure-time sports (OR: 1.98, 99 % CI: 1.05, 3.71 for mother-child pairs; OR: 2.83, 99 % CI: 1.52, 5.28 for father-child pairs), with no difference across years.

In North and South regions, the magnitude of parent-offspring associations for these behaviors also declined over time, similar to the Central region (data not shown). In Additional file 1: Table S3, we show the parent–child association by household income and geographic region at year 2000 for all dietary behaviors and at year 2004 for screen time. We found no interaction for leisure-time sports by any covariates; therefore leisure-time sports is not included in Additional file 1: Table S3.

Children’s diet and PA behaviors varied significantly by household structure (Table 3). Compared to children living with siblings, children without siblings consumed higher percentages of energy from all three categories. Only children were also more likely to participate in leisure-time sports than children living with siblings. Compared to children in households without grandparents, those living with grandparents consumed slightly less away-from-home foods.

**Table 3:** Predicted mean/probability (standard error) of diet, screen time, and PA in children by household structure^a^

	Has siblings	Only child	*P*	No grandparents	Has grandparents	*P*
Animal-source foods, % energy	12.0 (0.3)	13.4 (0.3)	<0.001	12.8 (0.3)	12.7 (0.3)	0.82
Away-from-home eating, % energy	21.1 (0.3)	25.0 (0.3)	<0.001	23.8 (0.3)	22.1 (0.3)	<0.001
Snacking, % energy	3.2 (0.7)	6.0 (1.2)	<0.001	5.1 (1.1)	6.0 (1.3)	0.21
Screen time, hrs/wk	13.3 (0.4)	14.1 (0.3)	0.11	14.2 (0.4)	13.5 (0.3)	0.12
Any leisure-time sports participation, %	26.4 (1.8)	32.9 (1.3)	0.005	32.5 (1.6)	29.1 (1.4)	0.13

^a^Separate random-effects negative binomial regression models for each behavior predicted adjusted mean values of animal-source foods, away-from-home eating, snacking, and screen time; random-effects logistic regression model predicted adjusted probability of leisure-time sports participation. All models controlled for child’s age (y) and sex, household income (tertiles), urbanicity (tertiles), geographic region (North/Central/South), year of study entry, and highest parental education (none or primary/middle school/high school/technical, college or higher). PA, physical activity

## Discussion

This longitudinal study provides insights into diet, screen time, and PA dynamics among children and their parents in rapidly modernizing China. Our findings suggest that over the follow-up, changes in behaviors over time differed between children and their parents. Despite the difference, overall we found positive parent-offspring associations for diet, screen time, and PA, although the magnitude of associations varied across behaviors. Higher intakes of animal-source food, away-from-home food, and snacks in parents were associated with higher intakes of these foods in their children. Children also spent more time on screen-based activities when their parents had higher screen time, and children were more likely to participate in leisure-time sports if their parents engaged in leisure-time sports. On the other hand, the magnitude of parent-offspring associations for these behaviors weakened over time for some but not all behaviors, suggesting differences in the face of the changing environment in China. We also observed statistically significant differences in diet and PA behaviors in children by household structure. Children with no siblings had higher intakes of animal-sources food, away-from-home food, and snacks compared to children who shared households with siblings.

We found statistically significant parent–child differences for the rate of changes in some, but not all, diet, screen time, and PA behaviors. Particularly, the increase in away-from-home eating and snacking was greater in children than in their parents in later years. Others have found some of these behavioral changes are linked with obesity [33]. A similar difference between offspring and maternal changes in obesogenic diet in response to urbanization was reported in a Filipino population [14]. This is possibly because children adjust more quickly to environmental changes than adults who are reluctant to accept changes [34]. Numerous food commercials targeting children as well as the increasing availability, accessibility, and affordability of meals and snacks at school may have also contributed to more rapid increase in intakes of away-from-home food and snacks in children than in adults. Our results support findings from acculturation studies where immigrant children were more open to adopting lifestyles in the new environment compared to their parents [35]. As more Westernized diets have been associated with higher risk of obesity [36, 37], the greater change in diet behaviors in children might partly explain the faster increase of obesity in children compared to adults in the past two decades in China [3].

Although similar diet, screen time, and PA behaviors between children and their parents seem to be apparent, Wang et al. have shown inconsistent parent-offspring relationships for diet in their meta-analysis [11]. The inconsistencies across studies may be due to differences in the study population, study time, diet and PA assessment tools, and analytical methods [11]. In our study, we found positive parent-offspring associations for diet, screen time, and PA behaviors. While previous studies mostly focused on intakes of certain food groups or nutrients when studying the parent–child resemblance in diet [7, 10, 38], we found positive parent-offspring associations for eating away-from-home and snacking. The magnitude of associations differed across behaviors but was stronger for snacking and leisure-time sports than other behaviors. A number of mechanisms may explain the observed parent–child associations for health behaviors. First, it is possible that parents act as role models for children, and children adopt their parent’s behavioral habits and attitudes. Second, shared household environment may have influenced both children’s and adults’ behaviors in similar ways. For example, studies have found positive associations between accessibility of facilities in the neighborhood (e.g., open space, recreational center, park) and participation in physical activity in both adults and children [39, 40]. Third, studies have shown that genetic predisposition may also contribute to the familial concordance of diet and PA behaviors [41, 42].

Despite the observed parent-offspring association for diet behaviors and screen time, the magnitude of these associations decreased over time, indicating possible differential influences of urbanization-related environmental changes in adults versus children. This is supported by our observed differential magnitude of parent-offspring associations for some of the behaviors by urbanicity, which indicates weaker associations in higher urbanicity areas. Increasing pocket money associated with the growing household income over time might have also led to less concordance in behaviors between children and their parents, particularly in diet, since children with extra pocket money are more likely to purchase meals or snacks on their own outside home.

In addition to the influence of parents, we found that children’s diet and PA behaviors also differed by household structure. This was particularly clear in the case of one-child family structure, which was associated with more modernized dietary behaviors in children relative to households with more than one child, independent of urbanicity and income levels of the household. The “One-Child Policy” implemented in 1979 resulted in a high proportion of single-child households. These only children often receive more attention and the best care in the household than children living with siblings [43]. As a compensation of their past experience of food shortage and deprivation, parents tend to indulge their only children with modern foods. The presence of grandparents in the household has also been associated with less healthy dietary habits in children, including larger portions of meals and higher consumption of unhealthy snacks [15–17]. This is due to grandparents’ desire to over-indulge their grandchildren and a belief that heavier children are healthier and children who eat more will grow taller. A previous study among Japanese children found an association between the presence of grandparents and physical inactivity [44]. In our research, children living with a grandparent had a lower probability of engaging in leisure-time sports, although the difference was not statistically significant. Unhealthy diet behaviors and physical inactivity may have contributed to the higher risk of overweight/obesity among children living in only-child and/or three-generation households relative to those who have siblings and live in two-generation households, as observed in previous studies [45–48]. Intervention strategies may focus on improving dietary habits and promoting PA to enhance the ability of improving health outcomes in children in such household situations.

Our study has some limitations. First, self-reported diet, screen time, and PA data may be subject to recall and social desirability biases, although the CHNS data are based on highly detailed recall methods, including analysis of household-specific recipes, weighing and measuring of condiments. Previous research on selected components of the diet (energy, protein, sodium, monosodium glutamate) suggest strong validity [49–52], and the PA components have been found to be highly predictive of incident obesity and weight gain in adults [27–30]. Second, loss to follow-up could have caused selection bias since we restricted to parent–child pairs who had at least two waves of diet, screen time, and PA data within the age range. We could have slightly underestimated the predicted values of some of the behaviors due to lower intakes of animal-sources food and away-from-home food, and lower levels of screen time in the included compared to the excluded samples. Third, for younger children who were under 10y, parents who reported their own diet, screen time, and PA and those of their children could have resulted in potential same-source bias and high parent–child correlation, especially for diet. However, we conducted sensitivity analyses to test whether our finding was robust to whether the child’s survey was aided by their parents (aged 7-10y only, 22 % total observations) and found similar pattern of changes and magnitude of estimated associations (not presented). Fourth, the lack of sampling weights in CHNS does not allow predicting population-relevant estimates of these behaviors. Finally, we cannot imply causation from these associations as it is impossible to unravel within-household dynamics.

In spite of its limitations, our study has several notable strengths. First, CHNS is one of the only population-based, longitudinal studies with diet and PA data collected at the household level over 20 years of rapid environmental change. Capitalizing on this unique dataset, we were able to determine and compare changes in diet, screen time, and PA in children and their parents during two decades in this rapidly modernizing country. Studying changes in health behaviors helps us understand the rapidly increasing prevalence of obesity and cardiometabolic risk factors in China in the past twenty years, whereas comparing the difference in changes between children and their parents provides insights into why urbanization-related increase of obesity is faster in children relative to adults [2]. Second, we collected detailed dietary behaviors from each member of the household, allowing us to examine additional diet behaviors besides nutrients or food intakes commonly assessed in previous studies. Third, using random-effects negative binomial regression models instead of simple correlation analysis, we were able to account for repeated measures over time within individuals.

## Conclusion

In summary, our findings suggest that changes in diet, screen time, and PA behaviors over the past two decades differed between children and parents in urbanizing China. Children’s behaviors were positively associated with parental behaviors, although the magnitude of the associations declined over time. Our work supports household-based versus individual-based health behavior interventions for both parents and children in promoting healthy dietary habits and increasing PA. However, generation-specific intervention strategies may be needed for children versus adults, especially for dietary behaviors, in household-based interventions due to the difference in changes of behaviors between generations with rapid environmental change. Household structure should also be considered for interventions targeting children’s behaviors in this population.

## Additional file

Additional file 1: Table S1. Baseline characteristics of children included and excluded in the analytic sample, CHNS 1991–2009. **Table S2.** Beta coefficients/odds ratios (OR) of diet, screen time, and PA among children, mothers, and fathers. **Figure S1.** Complete-case analysis: predicted mean (or probability) of diet, screen time, and PA over time. **Table S3.** Predicted parent-offspring association for diet, screen time, and PA by household income and geographic region. (DOCX 194 kb)

## Abbreviations

CHNS: China Health and Nutrition Survey
CI: Confidence interval
OR: Odds ratio
PA: Physical activity

## References

[1] Popkin BM (2001). Trends in diet, nutritional status, and diet-related noncommunicable diseases in China and India: the economic costs of the nutrition transition. Nutr Rev.

[2] Adair LS (2014). The emergence of cardiometabolic disease risk in Chinese children and adults: consequences of changes in diet, physical activity and obesity. Obes Rev.

[3] Popkin BM (2006). Is there a lag globally in overweight trends for children compared with adults?. Obesity.

[4] Weinsier RL (1998). The etiology of obesity: relative contribution of metabolic factors, diet, and physical activity. Am J Med.

[5] LaRosa J (1990). The cholesterol facts. A summary of the evidence relating dietary fats, serum cholesterol, and coronary heart disease. A joint statement by the American Heart Association and the National Heart, Lung, and Blood Institute. The Task Force on Cholesterol Issues, American Heart Association. Circulation.

[6] Sesso HD, Paffenbarger RS, Lee I-M (2000). Physical activity and coronary heart disease in men the Harvard Alumni Health Study. Circulation.

[7] Beydoun MA, Wang Y (2009). Parent–child dietary intake resemblance in the United States: evidence from a large representative survey. Soc Sci Med.

[8] Dearth-Wesley T (2011). Less traditional diets in Chinese mothers and children are similarly linked to socioeconomic and cohort factors but vary with increasing child age. J Nutr.

[9] Dearth-Wesley T (2012). Longitudinal, cross-cohort comparison of physical activity patterns in Chinese mothers and children. Int J Behav Nutr Phys Act.

[10] Mitchell BD (2003). Familial aggregation of nutrient intake and physical activity: results from the San Antonio Family Heart Study. Ann Epidemiol.

[11] Wang Y (2011). Do children and their parents eat a similar diet? Resemblance in child and parental dietary intake: systematic review and meta-analysis. J Epidemiol Community Health.

[12] Fuemmeler BF, Anderson CB, Mâsse LC (2011). Parent–child relationship of directly measured physical activity. Int J Behav Nutr Phys Act.

[13] Ornelas IJ, Perreira KM, Ayala GX (2007). Parental influences on adolescent physical activity: a longitudinal study. Int J Behav Nutr Phys Act.

[14] Kelles A, Adair L (2009). Offspring consume a more obesogenic diet than mothers in response to changing socioeconomic status and urbanization in Cebu, Philippines. Int J Behav Nutr Phys Act.

[15] Jingxiong J (2007). Influence of grandparents on eating behaviors of young children in Chinese three-generation families. Appetite.

[16] Li B, Adab P, Cheng KK (2015). The role of grandparents in childhood obesity in China-evidence from a mixed methods study. Int J Behav Nutr Phys Act.

[17] Roberts M, Pettigrew S (2010). The influence of grandparents on children’s diets. J Res Consumers.

[18] China Statistical Yearbook, National Bureau of statistics of China (2007). China Statistical Yearbook.

[19] Chen C (1996). Nutrition status of the Chinese people. Biomed Environ Sci.

[20] Ge K, Zhai F, Yan H (1996). The dietary and nutritional status of Chinese population (1992 National Nutrition Survey).

[21] Wang L (2005). Report of China nationwide nutrition and health survey 2002 (1): summary repo.

[22] Popkin BM (2010). The implications of the nutrition transition for obesity in the developing world.

[23] Zhai F (2014). Dynamics of the Chinese diet and the role of urbanicity, 1991–2011. Obes Rev.

[24] Yang Y (2005). Chinese food composition table 2004.

[25] Yao M (2003). Relative influence of diet and physical activity on cardiovascular risk factors in urban Chinese adults. Int J Obes Relat Metab Disord.

[26] He K (2011). Consumption of monosodium glutamate in relation to incidence of overweight in Chinese adults: China Health and Nutrition Survey (CHNS). Am J Clin Nutr.

[27] Bell AC, Ge K, Popkin BM. Weight gain and its predictors in Chinese adults. Int J Obes Relat Metab Disord. 2001;25(7):1079-86. 10.1038/sj.ijo.0801651 11443510

[28] Bell AC, Ge K, Popkin BM (2002). The road to obesity or the path to prevention: motorized transportation and obesity in China. Obes Res.

[29] Monda K (2008). Longitudinal relationships between occupational and domestic physical activity patterns and body weight in China. Eur J Clin Nutr.

[30] Ng SW (2012). Estimation of a dynamic model of weight. Empirical Economics.

[31] Jones-Smith JC, Popkin BM (2010). Understanding community context and adult health changes in China: development of an urbanicity scale. Soc Sci Med.

[32] Wang Z (2012). Trends in Chinese snacking behaviors and patterns and the social-demographic role between 1991 and 2009. Asia Pac J Clin Nutr.

[33] Zhou Y (2015). The food retail revolution in China and its association with diet and health. Food Policy.

[34] Kwak K (2003). Adolescents and their parents: A review of intergenerational family relations for immigrant and non-immigrant families. Hum Dev.

[35] Costigan CL, Dokis DP (2006). Similarities and differences in acculturation among mothers, fathers, and children in immigrant Chinese families. J Cross Cult Psychol.

[36] Astrup A (1994). Obesity as an adaptation to a high-fat diet: evidence from a cross-sectional study. Am J Clin Nutr.

[37] Popkin BM (2001). The nutrition transition and obesity in the developing world. J Nutr.

[38] Wang Y, Li J, Caballero B (2009). Resemblance in dietary intakes between urban low-income African-American adolescents and their mothers: the healthy eating and active lifestyles from school to home for kids study. J Am Diet Assoc.

[39] Davison KK, Lawson CT (2006). Do attributes in the physical environment influence children’s physical activity? A review of the literature. Int J Behav Nutr Phys Act.

[40] Humpel N, Owen N, Leslie E (2002). Environmental factors associated with adults’ participation in physical activity: a review. Am J Prev Med.

[41] Cleland V (2005). Parental exercise is associated with Australian children’s extracurricular sports participation and cardiorespiratory fitness: A cross-sectional study. Int J Behav Nutr Phys Act.

[42] Hur Y-M, Bouchard TJ, Eckert E (1998). Genetic and environmental influences on self-reported diet: a reared-apart twin study. Physiol Behav.

[43] Jing J. Feeding China's little emperors: Food, children, and social change. Stanford: Stanford University Press; 2000.

[44] Kagamimori S (1999). The relationship between lifestyle, social characteristics and obesity in 3‐year‐old Japanese children. Child Care Health Dev.

[45] Gopinath B (2012). Socio‐economic, familial and perinatal factors associated with obesity in Sydney schoolchildren. J Paediatr Child Health.

[46] Hunsberger M (2012). Overweight in singletons compared to children with siblings: the IDEFICS study. Nutrition & diabetes.

[47] Ochiai H (2012). Number of siblings, birth order, and childhood overweight: a population-based cross-sectional study in Japan. BMC Public Health.

[48] Watanabe E, Lee J, Kawakubo K (2011). Associations of maternal employment and three-generation families with pre-school children’s overweight and obesity in Japan. Int J Obes.

[49] Du S (2014). Understanding the patterns and trends of sodium intake, potassium intake, and sodium to potassium ratio and their effect on hypertension in China. Am J Clin Nutr.

[50] Paeratakul S (1998). Measurement error in dietary data: implications for the epidemiologic study of the diet-disease relationship. Eur J Clin Nutr.

[51] Popkin BM, Lu B, Zhai F (2002). Understanding the nutrition transition: measuring rapid dietary changes in transitional countries. Public Health Nutr.

[52] Zhai F (1996). Evaluation of the 24-hour individual recall method in China. Food and Nutrition Bulletin-United Nations University-.

