# Supplementary material for: Parent–child associations for changes in diet, screen time, and physical activity across two decades in modernizing China: China Health and Nutrition Survey 1991–2009
Source: Int J Behav Nutr Phys Act. 2016 Nov 11;13:118. doi: 10.1186/s12966-016-0445-z (PMC5106797; doi:10.1186/s12966-016-0445-z)
Supplement: Additional file 1: Table S1. — Baseline characteristics of children included and excluded in the analytic sample, CHNS 1991–2009. Table S2. Beta coefficients/odds ratios (OR) of diet, screen time, and PA among children, mothers, and fathers. Figure S1. Complete-case analysis: predicted mean (or probability) of diet, screen time, and PA over time. Table S3. Predicted parent-offspring association for diet, screen time, and PA by household income and geographic region. (DOCX 194 kb) [file 12966_2016_445_MOESM1_ESM.docx]

Additional file 1: Table S1. Baseline characteristics of children included and excluded in the analytic sample, CHNS 1991-2009^a^

|  | Included | Excluded | P-value |
| --- | --- | --- | --- |
| No. of subject | 5201 | 86 |  |
| Age, y (mean ± SD) | 10.2±2.5 | 10.3±2.4 | 0.62 |
| Gender, % male | 52.0 | 57.0 | 0.06 |
| Annual household income, 1000 yuan (mean ±SD)† | 15.2±18.4 | 20.2±22.2 | <0.001 |
| Urbanicity (mean ± SD)‡ | 47.9±17.7 | 56.4±19.7 | <0.001 |
| Geographic region, % |  |  | 0.013 |
| North | 15.2 | 10.1 |  |
| Central | 31.4 | 30.7 |  |
| South | 53.4 | 59.3 |  |
| Highest parental education, % |  |  | <0.001 |
| None/primary school | 10.3 | 8.4 |  |
| Middle school | 21.7 | 11.7 |  |
| High school | 61.8 | 69.2 |  |
| College, technical or higher | 6.3 | 10.8 |  |
| Animal-source food, % energy (mean ± SD) | 10.1±9.9 | 13.1±10.6 | <0.001 |
| Away-from-home eating, % energy (mean ± SD) | 20.5±17.0 | 28.3±22.1 | <0.001 |
| Snacking, % energy (mean ± SD) | 1.0±3.6 | 1.3±4.2 | 0.22 |
| Screen time, hours/week (mean ± SD) | 8.5±7.3 | 11.4±8.1 | <0.001 |
| Any leisure-time sports participation, % | 19.4 | 24.0 | 0.10 |
| Year of study entry, % |  |  | <0.001 |
| 1991 | 43.4 | 2.8 |  |
| 1993 | 9.5 | 1.3 |  |
| 1997 | 22.5 | 7.0 |  |
| 2000 | 12.1 | 9.5 |  |
| 2004 | 8.9 | 41.0 |  |
| 2006 | 3.7 | 38.4 |  |

^a^Difference tested using one-way ANOVA (continuous variables) or chi-squared test (categorical variables).

†Total household income inflated to 2011.

‡Urbanicity defined by a multicomponent urbanicity scale ranging from 0-120 [[22](#_ENREF_21)].

CHNS: China Health and Nutrition Survey

Additional file 1: Table S2. Beta coefficients/odds ratios (OR) of diet, screen time, and PA among children, mothers, and fathers^a^

|  | Animal-source foods, % energy | |  | Away-from-home  eating, % energy | |  | Snacking, % energy | |  | Screen time, hrs/wk | |  | Any leisure-time sports  participation, % | |
| --- | --- | --- | --- | --- | --- | --- | --- | --- | --- | --- | --- | --- | --- | --- |
|  | Beta | 99% CI |  | Beta | 99% CI |  | Beta | 99% CI |  | Beta | 99% CI |  | OR | 99% CI |
| 1991 | ref. | |  | ref. | |  | ref. | |  |  |  |  |  |  |
| 1993 | -0.01 | -0.11,0.08 |  | -0.05 | -0.13,0.03 |  | -0.95 | -1.51,-0.39 |  |  |  |  |  |  |
| 1997 | 0.05 | -0.05,0.15 |  | 0.18 | 0.09,0.26 |  | -0.67 | -1.26,-0.08 |  |  |  |  |  |  |
| 2000 | 0.40 | 0.30,0.51 |  | 0.31 | 0.23,0.40 |  | -1.36 | -2.00,-0.72 |  |  |  |  |  |  |
| 2004 | 0.40 | 0.28,0.52 |  | 0.42 | 0.31,0.52 |  | -1.58 | -2.33,-0.83 |  | ref. | |  | ref. | |
| 2006 | 0.60 | 0.47,0.73 |  | 0.35 | 0.24,0.46 |  | 0.37 | -0.39,1.13 |  | 0.18 | 0.07,0.30 |  | 1.53 | 1.16,2.03 |
| 2009 | 0.86 | 0.71,1.02 |  | 0.68 | 0.54,0.81 |  | 1.27 | 0.42,2.12 |  | 0.28 | 0.15,0.41 |  | 2.25 | 1.61,3.13 |
| Child | ref. | |  | ref. | |  | ref. | |  | ref. | |  | ref. | |
| Mother | 0.24 | 0.09,0.38 |  | 0.05 | -0.06,0.17 |  | -1.60 | -2.45,-0.76 |  | 0.54 | 0.30,0.78 |  | 0.06 | 0.02,0.21 |
| Father | 0.31 | 0.15,0.46 |  | 0.27 | 0.14,0.39 |  | -1.53 | -2.42,-0.63 |  | 0.45 | 0.20,0.70 |  | 0.03 | 0.01,0.13 |
| Mother X 1991 | - | - |  | ref. | |  | ref. | |  | - | - |  | - | - |
| Mother X 1993 | - | - |  | 0.00 | -0.09,0.09 |  | 0.81 | 0.15,1.47 |  | - | - |  | - | - |
| Mother X 1997 | - | - |  | -0.02 | -0.12,0.07 |  | 0.76 | 0.07,1.44 |  | - | - |  | - | - |
| Mother X 2000 | - | - |  | -0.02 | -0.12,0.07 |  | 0.73 | 0.03,1.43 |  | - | - |  | - | - |
| Mother X 2004 | - | - |  | -0.05 | -0.16,0.05 |  | 0.66 | -0.06,1.39 |  | ref. | |  | - | - |
| Mother X 2006 | - | - |  | -0.01 | -0.12,0.10 |  | 0.40 | -0.32,1.12 |  | -0.14 | -0.27,-0.01 |  | - | - |
| Mother X 2009 | - | - |  | -0.09 | -0.22,0.05 |  | 0.30 | -0.52,1.13 |  | 0.00 | -0.15,0.14 |  | - | - |
| Father X 1991 | - | - |  | ref. | |  | ref. | |  | - | - |  | - | - |
| Father X 1993 | - | - |  | 0.02 | -0.08,0.11 |  | 0.40 | -0.30,1.10 |  | - | - |  | - | - |
| Father X 1997 | - | - |  | -0.08 | -0.18,0.01 |  | 0.70 | -0.01,1.41 |  | - | - |  | - | - |
| Father X 2000 | - | - |  | -0.12 | -0.22,-0.03 |  | 0.92 | 0.19,1.64 |  | - | - |  | - | - |
| Father X 2004 | - | - |  | -0.12 | -0.23,-0.01 |  | -0.14 | -0.91,0.64 |  | ref. | |  | - | - |
| Father X 2006 | - | - |  | -0.13 | -0.25,-0.01 |  | -0.28 | -1.04,0.49 |  | -0.17 | -0.31,-0.04 |  | - | - |
| Father X 2009 | - | - |  | -0.21 | -0.34,-0.07 |  | -0.36 | -1.23,0.51 |  | -0.04 | -0.20,0.11 |  | - | - |
| Med. Urban X 1991 | ref. | |  | ref. | |  | ref. | |  | - | - |  | - | - |
| Med. Urban X 1993 | 0.17 | 0.05,0.30 |  | 0.15 | 0.06,0.24 |  | -0.26 | -0.94,0.43 |  | - | - |  | - | - |
| Med. Urban X 1997 | 0.25 | 0.11,0.38 |  | 0.08 | -0.02,0.17 |  | 0.11 | -0.58,0.80 |  | - | - |  | - | - |
| Med. Urban X 2000 | 0.06 | -0.07,0.20 |  | 0.20 | 0.11,0.30 |  | 0.27 | -0.45,0.99 |  | - | - |  | - | - |
| Med. Urban X 2004 | 0.05 | -0.09,0.20 |  | 0.22 | 0.12,0.32 |  | 1.96 | 1.18,2.74 |  | ref. | |  | - | - |
| Med. Urban X 2006 | 0.06 | -0.10,0.21 |  | 0.31 | 0.19,0.42 |  | 1.23 | 0.47,1.98 |  | -0.10 | -0.23,0.04 |  | - | - |
| Med. Urban X 2009 | -0.18 | -0.36,0.01 |  | 0.28 | 0.15,0.41 |  | 1.22 | 0.37,2.06 |  | -0.19 | -0.33,-0.04 |  | - | - |
| High Urban X 1991 | ref. | |  | ref. | |  | ref. | |  | - | - |  | - | - |
| High Urban X 1993 | 0.18 | 0.05,0.31 |  | 0.05 | -0.05,0.14 |  | 0.29 | -0.39,0.98 |  | - | - |  | - | - |
| High Urban X 1997 | 0.17 | 0.03,0.30 |  | -0.02 | -0.12,0.08 |  | 0.29 | -0.42,1.00 |  | - | - |  | - | - |
| High Urban X 2000 | -0.16 | -0.30,-0.02 |  | -0.08 | -0.18,0.02 |  | 0.53 | -0.19,1.26 |  | - | - |  | - | - |
| High Urban X 2004 | -0.22 | -0.37,-0.07 |  | -0.11 | -0.22,-0.00 |  | 2.15 | 1.37,2.94 |  | ref. | |  | - | - |
| High Urban X 2006 | -0.38 | -0.54,-0.22 |  | 0.03 | -0.08,0.15 |  | 1.02 | 0.26,1.78 |  | -0.05 | -0.18,0.09 |  | - | - |
| High Urban X 2009 | -0.62 | -0.81,-0.43 |  | -0.06 | -0.20,0.08 |  | 1.02 | 0.16,1.87 |  | -0.11 | -0.26,0.04 |  | - | - |

Due to our large sample size, 99% confidence intervals (CIs) were calculated instead of 95% CIs to correspond to the p-values of 0.01.

^a^Separate random-effects negative binomial regression models for each behavior of animal-source foods, away-from-home eating, snacking, and screen time; random-effects logistic regression models for leisure-time sports participation. All models controlled for age (y), household income (tertiles), urbanicity (tertiles), geographic region **(North/Central/South), and year of study entry.** PA, physical activity.

Additional file 1: Figure S1. Complete-case analysis: predicted mean (or probability) of diet, screen time, and PA over time
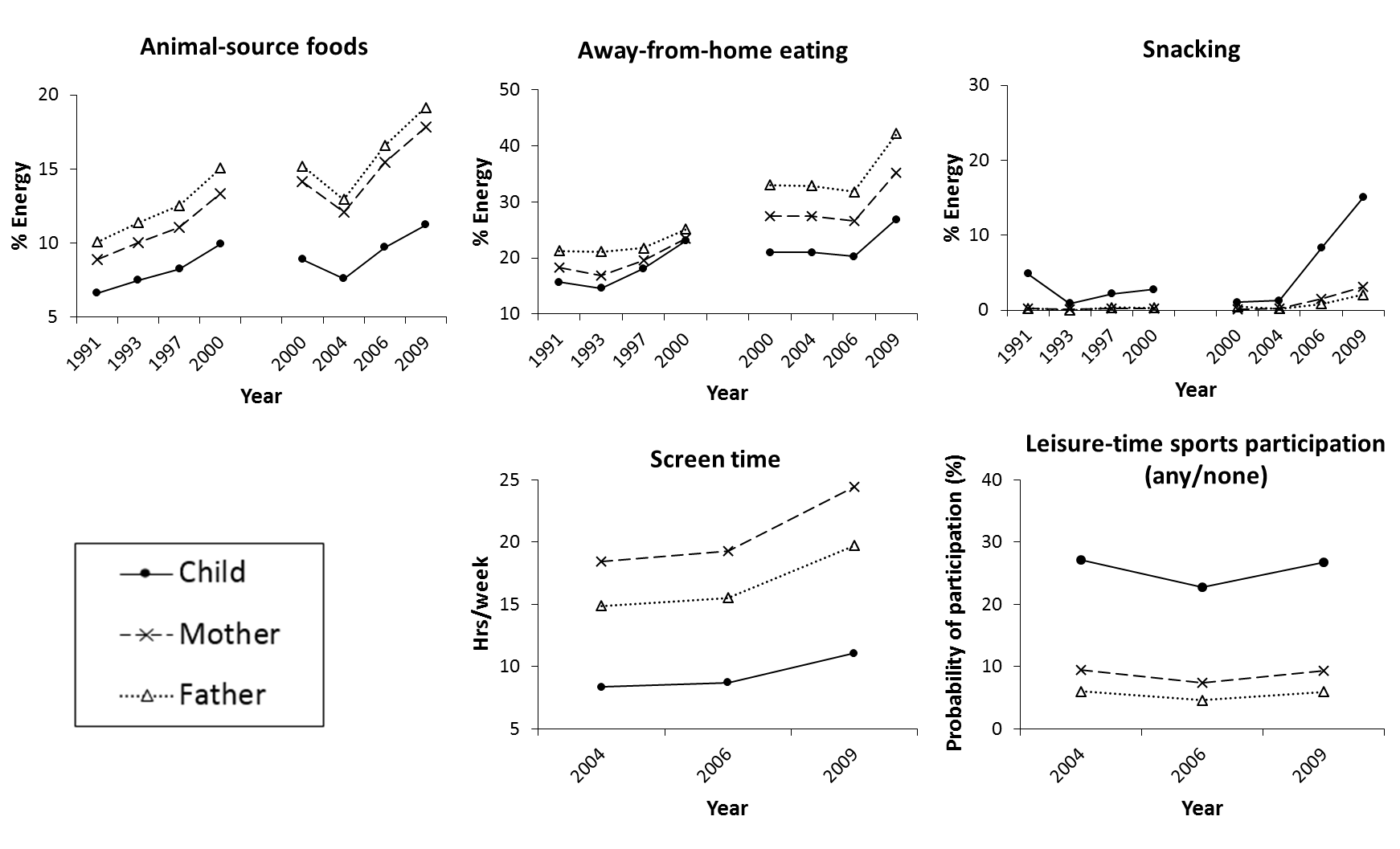


^a^We ran complete-case dietary analyses among two separate groups of individuals who completed all four surveys from 1991-2000 (n=1392), and from 2000-2009 (n=768), respectively, to compare the change of dietary behaviors over time with results from mixed groups of individuals in our main analysis who completed 2-4 surveys (Figure 1). For screen time and PA, we ran complete-case analyses among one group of individuals who completed all three surveys from 2004-2009 (n=1725), to compare the change of screen time and PA over time with results from mixed groups of individuals in our main analysis who completed 2-3 surveys. Separate random-effects negative binomial regression models for each behavior predicted adjusted mean values of animal-source foods, away-from-home eating, snacking, and screen time; random-effects logistic regression model predicted adjusted probability of leisure-time sports participation. All models controlled for baseline age (y), household income (tertiles), urbanicity (tertiles), geographic region **(North/Central/South), and year of study entry. The rate of changes in** away-from-home eating and snacking **was different across household members (p for interaction<0.01)**. PA, physical activity.

Additional file 1: Table S3. Predicted parent-offspring associations for diet, screen time, and PA by household income and geographic region^a^

|  | Animal-source food (% energy)^b^ | | | |  | Away-from-home food (% energy)^b^ | | | |  | Snacking (% energy)^b^ | | | |  | Screen time (hours/week)^c^ | | | |
| --- | --- | --- | --- | --- | --- | --- | --- | --- | --- | --- | --- | --- | --- | --- | --- | --- | --- | --- | --- |
|  | Mother | | Father | |  | Mother | | Father | |  | Mother | | Father | |  | Mother | | Father | |
|  | Beta | 99% CI | Beta | 99% CI |  | Beta | 99% CI | Beta | 99% CI |  | Beta | 99% CI | Beta | 99% CI |  | Beta | 99% CI | Beta | 99% CI |
| Household income^d^ |  |  |  |  |  |  |  |  |  |  |  |  |  |  |  |  |  |  |  |
| Low | 1.16 | 1.09,1.23 | 1.16 | 1.08,1.24 |  | 0.37 | 0.33,0.40 | 0.29 | 0.26,0.32 |  | n/a | n/a | n/a | n/a |  | n/a | n/a | n/a | n/a |
| Medium | 0.94 | 0.88,1.01 | 0.96 | 0.88,1.04 |  | 0.31 | 0.28,0.34 | 0.25 | 0.22,0.28 |  | n/a | n/a | n/a | n/a |  | n/a | n/a | n/a | n/a |
| High | 0.82 | 0.76,0.89 | 0.81 | 0.73,0.89 |  | 0.31 | 0.28,0.34 | 0.23 | 0.20,0.25 |  | n/a | n/a | n/a | n/a |  | n/a | n/a | n/a | n/a |
| Geographic region^e^ |  |  |  |  |  |  |  |  |  |  |  |  |  |  |  |  |  |  |  |
| North | 0.90 | 0.82,0.97 | 0.87 | 0.78,0.96 |  | 0.30 | 0.27,0.33 | 0.23 | 0.20,0.26 |  | 4.19 | 2.19,6.19 | 2.62 | 0.57,4.66 |  | 0.00 | -0.01,0.01 | 0.00 | -0.01,0.01 |
| Central | 0.94 | 0.88,1.01 | 0.96 | 0.88,1.04 |  | 0.31 | 0.28,0.34 | 0.25 | 0.22,0.28 |  | 5.51 | 3.45,7.56 | 3.44 | 1.49,5.39 |  | 0.01 | 0.00,0.02 | 0.01 | 0.00,0.02 |
| South | 0.76 | 0.70,0.82 | 0.80 | 0.73,0.87 |  | 0.26 | 0.24,0.29 | 0.20 | 0.17,0.22 |  | 5.67 | 3.77,7.57 | 4.46 | 2.59,6.34 |  | 0.01 | 0.00,0.02 | 0.02 | 0.01,0.03 |

Due to our large sample size, 99% confidence intervals (CIs) were calculated instead of 95% CIs to correspond to the p-values of 0.01.

^a^Table shows predicted associations in year 2000 due to statistically significant modification by year at p<0.01. Year 2000 was chosen as the mid-point of year 1991 to 2009. Predicted beta coefficients and 99% confidence intervals (CI) were estimated using separate random-effects negative binomial regression models for each behavior. n/a indicates no modification by household income (p for interaction>0.01); coefficients are the same across all household income levels in 2000 and are shown in Table 2 in the main text. All models controlled for child’s baseline age (y) and sex, household income (tertiles), urbanicity (tertiles), geographic region (North/Central/South), year of study entry, and highest parental education (none or primary/middle school/high school/technical, college or higher). PA, physical activity.

^b^Beta coefficients for animal-source foods, away-from-home eating, and snacking indicate the change of child’s daily intake in percentage of total energy when mother’s or father’s intake increased by 10% total energy.

^c^Beta coefficients for screen time indicate the change of child’s screen time in hours when mother’s or father’s screen time increased by one hour per week.

^d^Coefficients for household income were predicted for the Central region due to interactions between income with the behaviors, and between region with the behaviors.

^e^Coefficients for region were predicted at the medium income level due to interactions between income with the behaviors, and between region with the behaviors.
